# Enteropathic arthritis is associated with an increased risk of major adverse cardiovascular events and venous thromboembolism

**DOI:** 10.1093/rap/rkaf131

**Published:** 2025-11-13

**Authors:** Jacob C Williams, Phuong Le Kieu, Benjamin P Zuckerman, Uazman Alam, Sizheng Steven Zhao

**Affiliations:** NIHR Leeds Biomedical Research Centre, Leeds Teaching Hospitals NHS Trust, Leeds, UK; Leeds Institute of Rheumatic and Musculoskeletal Medicine, University of Leeds, Leeds, UK; Wythenshawe Hospital, Manchester University NHS Foundation Trust, Manchester, UK; Centre for Rheumatic Diseases, King’s College London, London, UK; Institute of Life Course and Medical Sciences, University of Liverpool, Liverpool, UK; Department of Medicine, University Hospital Aintree, Liverpool University NHS Foundation Trust, Liverpool, UK; Centre for Musculoskeletal Research, Division of Musculoskeletal and Dermatological Science, School of Biological Sciences, Faculty of Biological Medicine and Health, University of Manchester, Manchester Academic Health Science Centre, Manchester, UK; Rheumatology Department, Manchester University NHS Foundation Trust, Manchester, UK

**Keywords:** enteropathic arthritis, inflammatory bowel disease, spondyloarthritis, cardiovascular disease, venous thromboembolism

## Abstract

**Objectives:**

To assess the risk of major adverse cardiovascular events (MACE) and venous thromboembolism (VTE) in patients with enteropathic arthritis (EA) compared with matched controls.

**Methods:**

We performed a 1:1 propensity score matched retrospective cohort study using electronic health records. EA was defined using International Classification of Diseases, 10th Revision code M07 and codes for Crohn’s disease or ulcerative colitis, excluding other inflammatory arthritis. Controls had no coded diagnosis of Crohn’s disease, ulcerative colitis or inflammatory arthritis. Primary outcomes were MACE and VTE; secondary outcomes included myocardial infarction (MI), stroke, CVD (composite of ischaemic heart disease and cerebrovascular disease), pulmonary embolism (PE) and deep vein thrombosis (DVT). Cohorts were matched for demographics, comorbidities and medications, with analysis using Cox proportional hazards models.

**Results:**

We included 5239 matched pairs (mean age 43 years, 63% female), with follow-up of 19 256 person-years (PY) for EA and 42 064 PY for controls. MACE [261 events; incidence rate (IR) 13.6/1000 PY (95% CI 11.9, 15.2)] occurred more frequently in EA compared with controls [407 events; IR 9.7/1000 PY (95% CI 8.7, 10.6)]. Similarly, VTE occurred more frequently in the EA group, with 264 [IR 13.7/1000 PY (95% CI 12.1, 15.4)] compared with 250 events [IR 5.9/1000 PY (95% CI 5.2, 6.7)]. The hazards of MACE [HR 1.40 (95% CI 1.19, 1.66)] and VTE [HR 1.89 (95% CI 1.57, 2.27)] were significantly increased. Results were concordant across CVD, MI and PE, but lacked precision for stroke and DVT.

**Conclusion:**

EA is associated with an increased risk of MACE, VTE, MI, CVD and PE. Risk-reduction strategies and lifestyle measures should be clinical and research priorities.

Key messages

- Enteropathic arthritis (EA) appears to increase the risk of adverse cardiovascular events.
- People with EA have an increased risk of myocardial infarction.
- EA is associated with an increased risk of venous thromboembolism and pulmonary embolism.

## Introduction

Enteropathic arthritis (EA) is a member of the SpA family associated with IBD, which may affect the peripheral joints and axial skeleton [1–3]. In IBD, musculoskeletal involvement is the most common extraintestinal manifestation [1, 3–5]. For example, up to 40% of patients with IBD have musculoskeletal involvement, including up to 25% with spinal involvement, 35% with sacroiliitis and 35% with peripheral involvement [3]. The age of onset of EA is typically <45 years, with an equal sex preponderance [3, 6]. Clinical presentation is variable, including subacute peripheral oligoarthritis (type 1), chronic polyarthritis (type 2) and axial-predominant disease [2, 3, 6]. Despite its high prevalence in IBD, predisposition for working-age people and negative impact on function and quality of life, EA remains underresearched compared with other forms of SpA [2, 3].

An increased risk of cardiovascular events in SpA is well recognised, with a large meta-analysis demonstrating an increased risk of myocardial infarction [MI; risk ratio (RR) 1.52 (95% CI 1.29, 1.80)] and stroke [RR 1.21 (95% CI 1.00, 1.47)] when compared with the general population [7]. Venous thromboembolism (VTE) risk is elevated in axial SpA, with an RR of 1.60 (95% CI 1.05, 2.44) [8]. Similarly, meta-analyses in individuals with IBD demonstrate an increased risk of MI [HR 1.29 (95% CI 1.07, 1.56)], stroke [HR 1.15 (95% CI 1.09, 1.20)] and VTE [RR 2.03 (95% CI 1.72, 2.39)] [9, 10]. To our knowledge, no study to date has examined the risk of cardiovascular disease (CVD) or VTE in EA. Overall, CVD remains a leading cause of mortality in SpA and IBD [11–13]. The underlying risk factors responsible for an increased risk of cardiovascular and thromboembolic events are similar between SpA and IBD and include chronic systemic inflammation, metabolic syndrome and corticosteroid use [7, 8, 10–18]. Although EA shares a similar pathophysiology with IBD and SpA, the actual risks of CVD and VTE in EA are not well described.

Quantifying the risk of CVD and VTE is important in EA for several reasons. Establishing the frequency of these events in EA is critical for service planning and identifying those most at risk. Furthermore, if the risks of CVD and VTE are found to be significantly elevated, healthcare providers can reduce these risks through lifestyle measures and medications. Increased risks may also influence treatment decisions, particularly with Janus kinase inhibitors (JAKis), which are licensed for IBD but may be associated with an increased risk of CVD and VTE [2, 19].

We conducted a retrospective cohort study using electronic health records data to estimate the risk of CVD and VTE in patients with EA compared with controls.

## Methods

### Data source

We used electronic health records data from >30 countries, >150 healthcare organisations and >140 million individuals between March 2005 and March 2025. Records from North American (predominantly USA) secondary care and specialist centres form the majority of the database. Characteristics recorded in the database include patient demographics, coded diagnoses, current and past treatments and investigation results. Data were accessed from the TriNetX database. Additional ethical approval was not required because this study used only de-identified data and did not involve the collection, use or transmittal of individually identifiable data.

### Study participants

All participants were ≥18 years of age. EA was defined by at least one International Classification of Diseases, 10th Revision code M07 plus at least one code for either Crohn’s disease (K50) or ulcerative colitis (K51). Individuals with concurrent codes for AS (M45), RA (M05–06) or PsA (L40.5) were excluded to reduce the risk of misclassification, although we acknowledge that delineation of SpA subtypes is not always clear.

Controls are difficult to define within healthcare records. We identified controls as individuals with documentation of a general medical examination without any abnormal findings (Z00.0), excluding individuals with a code for EA, RA, AS, PsA or any non-infective inflammatory bowel disease (K50–52).

The index date was defined as the initial coded date for EA (M07) in the EA group or the date of general medical examination without any abnormal findings (Z00.0) in the control group. Individuals were required to have at least 12 months of health records data before the index date to allow covariate ascertainment and were followed up until the occurrence of the specified outcome, their last recorded medical encounter or the censoring date (25 March 2025).

### Outcomes

Our co-primary outcomes were MACE and VTE. MACE was a composite of MI (I21–24), cerebral infarction (I63) or the occurrence of a related cardiovascular or cerebrovascular procedure (e.g. percutaneous coronary intervention). VTE was defined as a composite of pulmonary embolism (PE; I26), deep vein thrombosis (DVT; I80.1, I80.2), other VTE (I80–82) or procedural codes for thrombolysis or thrombectomy. Secondary analyses were performed for the following outcomes separately: MI, stroke, any CVD [ischaemic heart disease (IHD; I20–25) or cerebrovascular disease (I60–I69)], PE and DVT. A full list of codes used to define each outcome is shown in Supplementary Table S1.

### Covariates

The following covariates were assessed within 12 months prior to the index date and included in the propensity score matched model: demographics (age, sex, race/ethnicity); comorbidities (IHD, cerebrovascular diseases, chronic obstructive pulmonary disease, overweight and obesity, type 2 diabetes mellitus, hyperlipidaemia, tobacco use/nicotine dependence, VTE, chronic kidney disease, heart failure, hypertension); medications (lipid-modifying agents, antihypertensives, diuretics, calcium channel blockers, beta-blocking agents, agents acting on the renin–angiotensin system, aspirin); CRP (as <5, 5–10, >10 mg/l or missing) levels and BMI (as <25, 25–30, >30 kg/m^2^ or missing). The full list of diagnostic, medication and procedural codes is provided in Supplementary Table S1. Individuals were required to have at least 1 year of data prior to the index date, thus there were no missing data for binary covariates.

### Statistical analysis

Incidence was calculated via Poisson approximation and presented as incidence per 1000 person-years (PY) and as incidence rate ratios (IRRs) comparing EA with controls, limited to those without a prior history of each outcome event.

One-to-one propensity score matching was used to balance covariates. Standardised mean difference (SMD) was used as a measure of satisfactory matching, where SMD <0.10 was adequate.

Propensity score matched Cox proportional hazards models were used to estimate HRs and 95% CIs for EA *vs* controls.

The following sensitivity analyses were added during peer review. First, we restricted the duration of follow-up to 3 years. Second, we added negative control outcomes to evaluate potential residual confounding—defined as an outcome that should not plausibly differ between individuals with EA and general population controls. There are no agreed upon or validated negative control outcomes in this context. We selected epilepsy (G40) and blindness/low vision (H54) based on the following criteria: they are not biologically caused by EA or its treatments, they are not strongly driven by key confounders and they occur with sufficient frequency to allow stable estimation. Demonstrating a null association with these outcomes would provide reassurance that any observed associations with our primary outcomes are less likely to be explained by unmeasured bias. Third, we repeated analyses after excluding individuals with instances of each specific outcome event prior to the index date. Statistical analysis was performed using the TriNetX platform.

## Results

We identified 5254 individuals with EA and 758 492 controls. The mean follow-up duration was 3.68 years (s.d. 2.84) for EA and 7.61 years (s.d. 3.61) for controls. Compared with controls, individuals with EA were predominantly female (63% *vs* 54.9%) and White (73.5% *vs* 65.7%). Individuals with EA had a higher prevalence of most comorbidities, notably IHD (2.3% *vs* 1.2%), chronic kidney disease (2.3% *vs* 0.6%) and cancer (13.1% *vs* 6.1%). The use of all medications, including antihypertensives (3.2% *vs* 0.8%), lipid-modifying drugs (9.5% *vs* 6.3%) and aspirin (3.0% *vs* 1.5%), was also higher in those with EA (Table 1).

**Table 1.** Baseline characteristics before and after propensity score matching

Characteristics	Before matching		After matching		SMD after matching
	EA	Controls	EA	Controls	
Patients, *n*	5254	758 492	5239	5239	N/A
Age, years, mean (s.d.)	43.3 (17.6)	45.0 (17.7)	43.3 (17.6)	43.9 (17.8)	0.034
White	73.5	65.7	73.5	74.9	0.032
Female	63.0	54.9	63.0	62.2	0.016
Ischaemic heart diseases	2.3	1.2	2.3	2.3	0.001
Cerebrovascular diseases	1.0	0.7	1.0	1.0	0.01
Chronic obstructive pulmonary disease	1.1	0.5	1.1	1.1	0.002
Overweight and obesity	7.2	2.2	7.1	7.3	0.006
Type 2 diabetes mellitus	3.7	3.5	3.7	3.8	0.006
Disorders of lipoprotein metabolism and other lipidaemias	10.2	13.1	10.2	11.1	0.029
Tobacco use	0.8	0.3	0.8	0.8	0.002
Nicotine dependence	4.2	1.3	4.2	3.9	0.014
Other venous embolism and thrombosis	1.6	0.3	1.6	1.4	0.017
Chronic kidney disease	2.3	0.6	2.3	2.0	0.016
Neoplasms	13.1	6.1	13.0	12.4	0.019
Heart failure	1.3	0.4	1.2	1.0	0.024
Hypertension	13.2	10.3	13.2	13.6	0.012
Pulmonary embolism	0.7	0.2	0.7	0.5	0.025
Lipid-modifying agents	9.5	6.3	9.5	10.1	0.021
Antihypertensives	3.2	0.8	3.2	3.0	0.011
Diuretics	6.7	4.4	6.7	6.9	0.009
Calcium channel blockers	4.7	2.2	4.7	5.0	0.015
Beta blockers	8.4	3.6	8.3	8.8	0.016
Agents acting on the renin-angiotensin system	6.7	5.4	6.7	7.4	0.028
Aspirin	3.0	1.5	3.0	3.2	0.01
CRP, mg/l, mean (s.d.)	18.2 (36.5)	12.4 (32.4)	18.2 (36.5)	23.8 (51.7)	0.125
BMI, kg/m^2^, mean (s.d.)	27.4 (6.8)	28.7 (6.7)	27.4 (6.8)	28.0 (7.3)	0.095

Values are in percentages unless stated otherwise.

CRP data missing in 88% and BMI in 40% of matched populations.

Following propensity score matching, 5239 individuals were included in each group, with a mean follow-up of 3.68 years (s.d. 2.84) and 8.03 years (s.d. 3.32) for EA and controls, respectively. Covariates were well balanced (SMD <0.1), except for CRP (Table 1).

In matched populations, MACE [261 events; incidence rate (IR) 13.6/1000 PY (95% CI 11.9, 15.2)] occurred more frequently in EA compared with controls [407 events; IR 9.7/1000 PY (95% CI 8.7, 10.6)]. Similarly, VTE occurred more frequently in the EA group, with 264 events [IR 13.7/1000 PY (95% CI 12.1, 15.4)] compared with 250 events [IR 5.9/1000 PY (95% CI 5.2, 6.7)]. The incidence of primary outcomes was similar in the unmatched data, and the incidence of all secondary outcomes was higher in EA compared with controls in the matched and unmatched populations (Supplementary Tables S2 and S3).

The hazard of cardiovascular events was significantly higher in the EA group, including for MACE [HR 1.40 (95% CI 1.19, 1.66)] and CVD [HR 1.38 (95% CI 1.21, 1.57)]. The increased hazard of MACE appears to be largely driven by MI [HR 1.86 (95% CI 1.39, 2.49)], with no significant difference in stroke hazard detected [HR 1.14 (95% CI 0.84, 1.54)].

**Figure 1.**
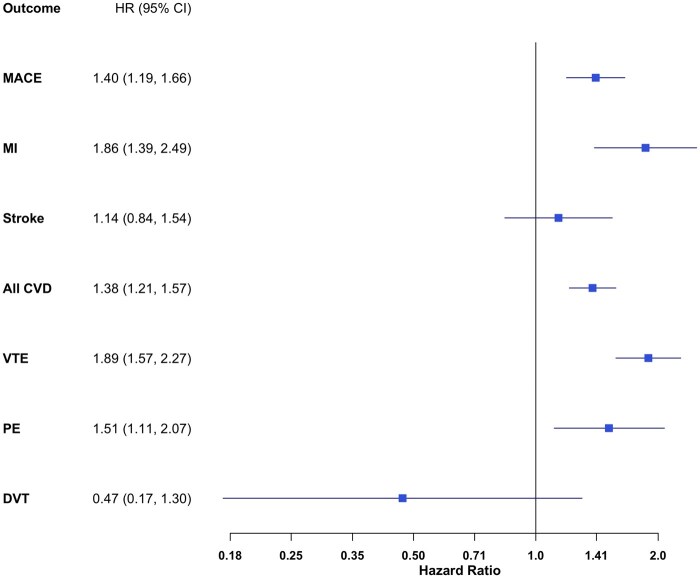
Risk of cardiovascular and thromboembolic outcomes in EA compared with matched controls

The hazard of VTE [HR 1.89 (95% CI 1.57, 2.27)] and PE [HR 1.51 (95% CI 1.11, 2.07)] were significantly increased in the EA cohort, with no difference for DVT [HR 0.47 (95% CI 0.17, 1.30)] (see Fig. 1).

**Figure 2.**
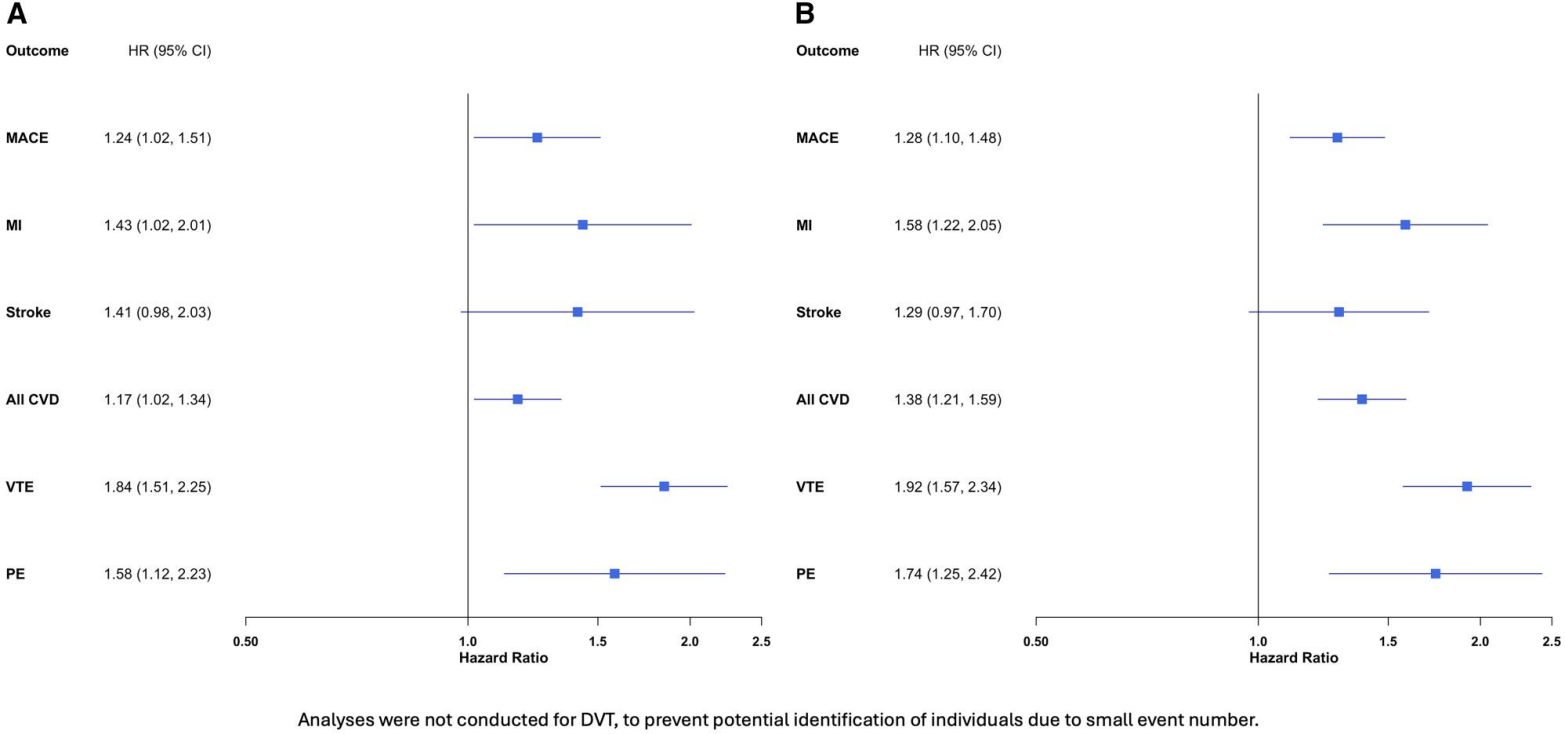
Sensitivity analysis with **(A)** follow-up restricted to 3 years and **(B)** individuals with pre-index outcome events excluded

Estimates from sensitivity analyses that restricted follow-up to 3 years and excluded individuals with pre-index outcome events were consistent with the main results, although CIs were wider due to a smaller sample size and fewer outcome events (Fig. 2). There was no association between EA and epilepsy [80 events; HR 1.04 (95% CI 0.76, 1.41)] or EA and blindness/low vision [43 events; HR 0.99 (95% CI 0.66, 1.49)].

## Discussion

In this matched study of the largest cohort of EA to date, we found that EA was associated with an increased hazard of MACE and VTE compared with controls. Results were concordant across CVD, MI and PE as secondary outcomes, although stroke and DVT lacked precision.

Our results were not unexpected. The risk of CVD and VTE has not been described in EA. However, an increased risk of CVD and VTE in IBD and SpA has been described [7–10, 20]. A retrospective study by Chan et al. [20] demonstrated the hazard of MACE was elevated [HR 1.70 (95% CI 1.29, 2.26)] in SpA and a large meta-analysis showed an increased risk of MI [RR 1.52 (95% CI 1.29, 1.80)] and stroke [RR 1.21 (95% CI 1.0–1.47)] [7]. In IBD, the hazards of MACE [HR 1.19 (95% CI 1.09, 1.30)], MI [HR 1.29 (95% CI 1.07, 1.56)] and stroke [HR 1.15 (95% CI 1.09, 1.20)] were all elevated [9]. An increased risk of VTE in EA also aligns with previous studies in axial SpA [RR 1.60 (95% CI 1.05, 2.44)] and IBD [RR 2.03 (95% CI 1.72, 2.39)] [8, 10]. Due to differences in population and study design, we are unable to directly compare our findings with those reported in previous studies.

The mechanisms underlying increased CVD and VTE hazards in EA are likely similar to those in other forms of SpA and IBD. Chronic inflammation accelerates atherosclerosis and contributes to the development of CVD [3, 15, 16, 21–23]. Relevant comorbidities, including hypertension, type 2 diabetes and hyperlipidaemia, are more common in psoriatic arthritis and axial SpA and are associated with the development of CVD [15, 16, 24]. Comorbidities in EA have not been well described in the literature; however, in our cohort, we demonstrated higher rates of hypertension, overweight/obesity and chronic kidney disease compared with controls. JAKis, which are licensed for use in IBD, are associated with an increased risk of both CVD and VTE in patients with RA [19]. However, studies in SpA and IBD have yet to demonstrate an increased cardiovascular risk in these patients and the risks of JAKis in EA remain unclear [19, 25, 26]. Finally, lifestyle factors, including diet, exercise and smoking, may also modify risk. Notably, musculoskeletal symptoms can be a barrier to exercise in axial SpA and could also be a contributing factor in EA [27].

In clinical practice, CVD is increasingly recognised and addressed in both SpA and IBD [23, 28]. Our results suggest that similar attention is warranted in EA, with a particular focus on modifying risk factors. Smoking is associated with radiographic progression of EA, and smoking and obesity are associated with reduced TNF inhibitor response [29–31]. Exercise has been demonstrated to help improve disease activity in SpA over the long term [32, 33]. Interventions such as smoking cessation, lipid management, exercise and weight loss should form key components of the holistic management of those with EA. Future studies should directly compare CVD and VTE risk in EA with that in other forms of SpA and IBD alone to better define the relative risk of these outcomes between diseases and guide screening and prevention strategies.

Our study has several strengths. It is the largest analysis to date exploring the hazards of CVD and VTE in EA. Furthermore, strict inclusion and exclusion criteria were applied, requiring coded diagnoses of IBD, but no coded diagnosis of any other inflammatory arthritides, which minimised misclassification bias.

However, our study also has limitations. Although we made efforts to reduce misclassification by using carefully defined groups, the use of coded data means misclassification remains possible. We were unable to apply previously validated definitions of venous thromboembolism that combine diagnostic codes with prescription data, and therefore some degree of outcome misclassification is possible. However, such misclassification would be expected to be non-differential between cases and controls. To our knowledge, no prior studies have validated codified definitions of EA, and therefore we were unable to provide a positive predictive value for our case definition. More research is needed to validate EA definitions. Residual confounding remains possible due to poorly captured variables, including disease activity and exercise levels. Importantly, we were unable to account for differences in medications that may contribute to outcome risk; e.g. it was not possible to match for glucocorticoid use because the presence of this medication in a non-disease population would be negligible. Because our controls were hospital-based, they may not fully represent the general population and are likely to include a higher burden of comorbidity. This would be expected to bias our estimates towards the null (as cardiovascular risk in the general population would likely be lower than in our control group) and should be taken into account when interpreting the findings. We propensity matched individuals across a 20-year period, which raises the possibility that some matched pairs were drawn from different calendar times when cardiovascular prevention and care may have differed. However, given the very large pool of eligible controls from which matches were selected, we consider this source of confounding to be unlikely. Future studies could additionally match for index date, which was not possible herein. Balancing CRP between patients with EA and a general ‘healthy’ control population is inherently problematic, which likely explains the higher SMD post-matching. For BMI, the SMD was close to 0.10 and the mean BMI remained slightly higher in controls. Since higher BMI is associated with increased cardiovascular risk, this residual imbalance would be expected to bias effect estimates towards the null. Despite the large sample size, the number of stroke and DVT events was low, making the null findings possibly attributable to type II error. Finally, death registry data were unavailable, meaning outcomes recorded solely as a cause of death were not reported, which may have underestimated fatal events. However, given that CVD is a leading cause of death, the absence of cardiovascular deaths from our outcome definition would be expected to reduce statistical power rather than introduce bias, likely biasing estimates towards the null.

In conclusion, our results demonstrate an increased hazard of MACE and VTE in patients with EA, underscoring the importance of cardiovascular risk reduction in this population. Future studies should seek to validate these findings, explore the underlying mechanisms, identify differences by age and sex and compare the relative risk of MACE and VTE in EA with that in RA, other SpA subtypes and IBD.

## Supplementary Material

rkaf131_Supplementary_Data

## Data Availability

The data supporting the findings of this study are available from TriNetX; however, third-party restrictions apply to the availability of these data. The data were used under license for this study with restrictions that do not allow for the data to be redistributed or made publicly available. However, for accredited researchers, the TriNetX data are available for licensing at TriNetX. To access the data in the TriNetX research network, a request can be submitted to TriNetX (trinetx.com). However, costs may be incurred, a data sharing agreement is necessary and no patient-identifiable information can be obtained.
