# Supplementary material for: Enteropathic arthritis is associated with an increased risk of major adverse cardiovascular events and venous thromboembolism
Source: Rheumatol Adv Pract. 2025 Nov 13;9(4):rkaf131. doi: 10.1093/rap/rkaf131 (PMC12688473; doi:10.1093/rap/rkaf131)
Supplement: rkaf131_Supplementary_Data [file rkaf131_supplementary_data.docx]

Supplementary Table S1. Codes used to define population, covariates and outcomes.

| Category | Variable | ICD-10, CPT, SNOMED or RxNorm code |
| --- | --- | --- |
| Inclusion | Enteropathic arthropathies | M07 |
| Inclusion | Crohn’s disease | K50 |
| Inclusion | Ulcerative colitis | K51 |
| Inclusion (control) | Encounter for general adult medical examination | Z00.0 |
| Exclusion | Ankylosing spondylitis | M45 |
| Exclusion | Rheumatoid arthritis with rheumatoid factor | M05 |
| Exclusion | Other rheumatoid arthritis | M06 |
| Exclusion | Arthropathic psoriasis | L40.5 |
| Exclusion | Noninfective enteritis and colitis | K50–K52 |
| Outcomes | MACE (MI or Stroke) | I21–I24, I61, I63, CPT1006199, CPT1006207, CPT1006216, CPT1021163-CPT1021168, CPT61645, CPT37195, CPT1012986 |
| Outcomes | Myocardial infarction (MI) | I21–I24, CPT1006199, CPT1006207, CPT1006216, CPT1021163-CPT1021168, CPT1012986 |
| Outcomes | Stroke | I61, I63, CPT61645, CPT37195 |
| Outcomes | All CVD | I20–I25, I60–I69 |
| Outcomes | VTE | I26, I80–I82, SNOMED276985008, SNOMED233428003, SNOMED 429141007, SNOMED 2977001, SNOMED 22276002, SNOMED 405315005 |
| Outcomes | PE | I26 |
| Outcomes | DVT | I80.1, I80.2, SNOMED2977001  SNOMED405315005 |
| Negative control outcomes | Epilepsy | G40 |
| Negative control outcomes | Blindness/low vision | H54 |
| Covariates | Ischemic heart diseases | I20–I25 |
| Covariates | Cerebrovascular diseases | I60–I69 |
| Covariates | Heart failure | I50 |
| Covariates | Hypertension | I10 |
| Covariates | Overweight/obesity | E66 |
| Covariates | Type 2 diabetes | E11 |
| Covariates | Disorders of lipoprotein metabolism and other lipidemias | E78 |
| Covariates | Chronic obstructive pulmonary disease (COPD) | J44 |
| Covariates | Tobacco use | Z72.0 |
| Covariates | Nicotine dependence | F17 |
| Covariates | Mood [affective] disorders | F30–F39 |
| Covariates | Chronic kidney disease (CKD) | N18 |
| Covariates | Neoplasms | C00–D49 |
| Medications | Lipid modifying agents | C10 |
| Medications | Antihypertensives | C02 |
| Medications | Diuretics | C03 |
| Medications | Calcium channel blockers | C08 |
| Medications | Beta blocking agents | C07 |
| Medications | Agents acting on the renin-angiotensin system | C09 |
| Medications | Aspirin | 1191 |

Supplementary Table S2. Crude incidence rates of key outcomes in unmatched populations.

| Outcome | EA | | | Controls | | | IRR (95% CI) |
| --- | --- | --- | --- | --- | --- | --- | --- |
|  | Events | PY | IR/1000PY (95% CI) | Events | PY | IR/1000PY (95% CI) |  |
| MACE | 250 | 18978 | 13.2 (11.5, 14.8) | 60,897 | 6363555 | 9.57 (9.49, 9.65) | 1.38 (1.22, 1.56) |
| MI | 90 | 18978 | 4.7 (3.8, 5.7) | 15,465 | 6363555 | 2.43 (2.39, 2.47) | 1.95 (1.59, 2.40) |
| Stroke | 71 | 18978 | 3.7 (2.9, 4.6) | 16,653 | 6363555 | 2.62 (2.58, 2.66) | 1.43 (1.13, 1.80) |
| All CVD | 427 | 18978 | 22.5 (20.4, 24.6) | 79,453 | 6363555 | 12.49 (12.40, 12.57) | 1.80 (1.64, 1.98) |
| VTE | 262 | 18978 | 13.8 (12.1, 15.5) | 25,429 | 6363555 | 4.00 (3.95, 4.05) | 3.45 (3.06, 3.90) |
| PE | 83 | 18978 | 4.4 (3.4, 5.3) | 8,242 | 6363555 | 1.30 (1.27, 1.32) | 3.38 (2.72, 4.19) |
| DVT | 10 | 18978 | 0.5 (0.2, 0.9) | 1,283 | 6363555 | 0.20 (0.19, 0.21) | 2.61 (1.40, 4.87) |
| CVD = cardiovascular disease, DVT = deep vein thrombosis, EA = enteropathic arthritis, IR = incidence rate, IRR = incidence rate ratio, MACE = major adverse cardiovascular events, MI = myocardial infarction, PE = pulmonary embolism, PY = patient-years, VTE = venous thromboembolism. | | | | | | | |

Supplementary Table S3. Incidence rates in matched populations.

| Outcome | EA | | | Controls | | | IRR (95% CI) |
| --- | --- | --- | --- | --- | --- | --- | --- |
|  | Events | PY | IR/1000PY (95% CI) | Events | PY | IR/1000PY (95% CI) |  |
| MACE | 261 | 19256 | 13.6 (11.9, 15.2) | 407 | 42064 | 9.7 (8.7, 10.6) | 1.40 (1.20, 1.64) |
| MI | 98 | 19256 | 5.1 (4.1, 6.1) | 110 | 42064 | 2.6 (2.1, 3.1) | 1.95 (1.48, 2.56) |
| Stroke | 72 | 19256 | 3.7 (2.9, 4.6) | 129 | 42064 | 3.1 (2.5, 3.6) | 1.22 (0.91, 1.63) |
| All CVD | 434 | 19256 | 22.5 (20.4, 24.7) | 561 | 42064 | 13.3 (12.2, 14.4) | 1.69 (1.49, 1.92) |
| VTE | 264 | 19256 | 13.7 (12.1, 15.4) | 250 | 42064 | 5.9 (5.2, 6.7) | 2.31 (1.94, 2.74) |
| PE | 81 | 19256 | 4.2 (3.3, 5.1) | 93 | 42064 | 2.2 (1.8, 2.7) | 1.90 (1.41, 2.56) |
| DVT | 10 | 19256 | 0.5 (0.2, 0.8) | 17 | 42064 | 0.4 (0.2, 0.6) | 1.29 (0.59, 2.81) |
| CVD = cardiovascular disease, DVT = deep vein thrombosis, EA = enteropathic arthritis, IR = incidence rate, IRR = incidence rate ratio, MACE = major adverse cardiovascular events, MI = myocardial infarction, PE = pulmonary embolism, PY = patient-years, VTE = venous thromboembolism. | | | | | | | |
